# Recombinant Attenuated *Edwardsiella piscicida* Vaccine Displaying Regulated Lysis to Confer Biological Containment and Protect Catfish against Edwardsiellosis

**DOI:** 10.3390/vaccines11091470

**Published:** 2023-09-09

**Authors:** Banikalyan Swain, Vanessa A. Campodonico, Roy Curtiss

**Affiliations:** Department of Infectious Diseases & Immunology, College of Veterinary Medicine, University of Florida, Gainesville, FL 32608, USA

**Keywords:** fish vaccine, immunity, pathogenicity, *E. piscicida*, *murA*, TLR & NLR pathway

## Abstract

We implemented a unique strategy to construct a recombinant attenuated *Edwardsiella* vaccine (RAEV) with a biological containment phenotype that causes regulated bacterial cell wall lysis. This process ensures that the vaccine strain is not able to persist in the environment. The *murA* gene is responsible for the catalysis of one of the first steps in the biosynthesis of muramic acid, which is a crucial component of the bacterial cell wall. The regulated lysis phenotype was achieved by inserting the tightly regulated *araC* P_araBAD_ cassette in place of the chromosomal *murA* promoter. Strains with this mutation require growth media supplemented with arabinose in order to survive. Without arabinose, they are unable to synthesize the peptidoglycan cell wall. Following the colonization of fish lymphoid tissues, the *murA* protein is no longer synthesized due to the lack of arabinose. Lysis is subsequently achieved in vivo, thus preventing the generation of disease symptoms and the spread of the strain into the environment. Vaccine strain χ16016 with the genotype ΔP_murA180_::TT *araC* P_araBAD_ *murA* is attenuated and shows a higher LD_50_ value than that of the wild-type strain. Studies have demonstrated that χ16016 induced TLR4, TLR5, TLR8, TLR9, NOD1 and NOD2-mediated NF-κB pathways and upregulated the gene expression of various cytokines, such as *il-8*, *il-1β*, *tnf-a*, *il-6* and *ifn-γ* in catfish. We observed significant upregulation of the expression profiles of *cd4*, *cd8* and *mhc-II* genes in different organs of vaccinated catfish. Vaccine strain χ16016 induced systemic and mucosal IgM titers and conferred significant protection to catfish against *E. piscicida* wild-type challenge. Our lysis RAEV is the first live attenuated vaccine candidate designed to be used in the aquaculture industry that displays this biological containment property.

## 1. Introduction

The global aquaculture industry is quickly becoming a major contributor to the farming economy. The need for reasonably priced and high-quality animal protein is increasing in tandem with the human population. Catfish are one of the most prominent aquaculture species farmed in the United States, with sales adding up to $421 million in 2021 (12% increase compared to 2020). Mississippi, Alabama, Arkansas and Texas account for 97% of these sales, making these states some of the most prominent producers of catfish in the United States [1].

In catfish production, one of the primary limiting factors is disease outbreaks [2]. Infectious diseases are responsible for a significant percentage of losses, with around 65% of fry and fingerlings being lost due to disease [3]. Among the most common of these diseases are enteric septicemia, columnaris disease and edwardsiellosis, which are brought on by *Edwardsiella ictaluri*, *Flavobacterium columnare* and *Edwardsiella piscicida*, respectively [4]. The infectious nature of these diseases facilitates large-scale outbreaks and has resulted in significant economic losses in the aquafarming industry. Several species derived from the genus *Edwardsiella* are known to pose a threat to catfish aquaculture. Among these species, *E. ictaluri*, *E. tarda* and *E. piscicida* are the most important threats, being responsible for massive economic losses in cultured catfish farming worldwide [5]. Isolates from infected farm-raised catfish in Mississippi previously thought to be *E. tarda* have been revealed to be *E. piscicida*, indicating that the increase in the prevalence of *E. tarda* infections was actually an increase in *E. piscicida* infections [6]. Enteric septicemia of catfish brought on by *Edwardsiella* species can result in nervous system impairment due to encephalitis, petechial hemorrhaging, distended abdomen, etc. This disease is highly transmissible: infected catfish can spread the bacteria into the water through their feces, enabling otherwise healthy fish to ingest it during feeding [7]. The overuse of antibiotics to treat infections caused by bacterial pathogens has been associated with the increasing prevalence of antibiotic-resistant bacteria, which have the potential to infect humans (via the consumption of infected fish) [8]. 

Vaccination is an extremely powerful tool; it is an alternative to using antibiotics in the prevention of infectious diseases and has the potential to enhance production yields. Live recombinant attenuated *Edwardsiella piscicida* vaccine (RAEV) strains can induce protective immunity against edwardsiellosis in fish [9]. Live attenuated bacterial vaccines tend to induce a more potent immune response than inactivated vaccines; they can elicit the same type of antigenic stimulation that would be expected following natural exposure to said pathogen without causing serious disease [10].

In constructing an effective live attenuated vaccine, the goal of this study is to alter the microbe’s genome in such a way that its pathogenicity is minimized but it is still able to induce an immune response [11]. Traditional in-frame gene deletion can result in the over-attenuation of the bacterial strain, impeding adequate host tissue colonization and increasing susceptibility to host defenses. We can avoid this issue by employing a regulated delayed -attenuation system, given that phenotypic expression is dependent on the availability of some externally supplied nutrients. Replacing the *fur* and *crp* gene promoters in *E. piscicida* with a highly regulated *araC* P_araBAD_ cassette makes it clear that expression is dependent on the presence of arabinose. Following vaccination, the strain will appear to elicit the same type of virulence expected from the wild type, but the absence of arabinose within the host tissue allows gradual attenuation and the prevention of full-blown infection [9]. 

*MurA* encodes an enzyme responsible for the catalysis of a crucial step in the synthesis of bacterial peptidoglycan [12]. Peptidoglycan is a component of the bacterial cell wall; it plays a role in ensuring the structural integrity of the cell and providing protection against environmental stressors. *MurA* is often a target when devising antibacterial strategies because its deletion is fatal to bacteria [13]. Several recombinant attenuated *Salmonella* vaccines (RASVs) employ deletion–insertion mutations that place certain genes, like *murA*, under the transcriptional control of the *araC* P_araBAD_ promoter, making it clear that they are not expressed in the absence of arabinose. Following host tissue invasion, the lack of arabinose within host tissue results in the cessation of the transcription of these genes and a decrease in their gene products with each cell division thereafter. The incapacity to transcribe *murA* allows programmed cell lysis and the subsequent prevention of environmental shedding [14]. 

*Edwardsiella* and *Aeromonas* spp. infections have become increasingly prevalent in US catfish aquaculture, especially in channel catfish (*Ictalurus punctatus*) [15,16]. Live recombinant immersion vaccines, which protect against a variety of diseases by expressing multiple protective antigens at a low cost, have not yet been developed for use in the aquaculture industry. Our efforts are, therefore, directed at addressing this need to develop safe efficacious vaccines that would be cost-effective to manufacture and administer. The acquisition of antibiotic resistance by the aforementioned bacteria has prompted fish farmers to look at vaccination as an alternative to the use of antibiotics for the treatment of these diseases [17]. 

The purpose of this study is to design, construct and evaluate a safe efficacious and mucosally delivered recombinant attenuated *Edwardsiella piscicida* vaccine (RAEV) to prevent outbreaks caused by *Edwardsiella* spp. in the aquaculture industry, thus increasing the sustainability and profitability of the global finfish aquaculture industry. This vaccine construct is sensitive to all antibiotics and designed to exhibit programmed lysis features to enable them to lyse after efficiently colonizing host lymphoid tissues. None of the bacterial vaccine cells are able to survive and, thus, exhibit complete biological containment. The strain was then evaluated for its ability to elicit innate and adaptive immune responses and its efficacy in terms of protecting catfish against edwardsiellosis.

## 2. Materials and Methods

### 2.1. Bacterial Strains, Plasmids and Growth Conditions

The bacterial strains and plasmids used in this study are listed in Table 1. *Escherichia coli* and *E. piscicida* derivative strains were routinely cultured in Luria–Bertani (LB) agar or LB broth. Media was supplemented with colistin sulfate (Col) (12.5 µg/mL), chloramphenicol (Cm) (25 µg/mL), 10% sucrose, arabinose (0.2%) and 50 μg/mL of diaminopimelic acid (DAP) when necessary. Bacterial growth was measured via the plating of serially diluted cultures and spectrophotometry. Oligonucleotides were sourced from IDT (Coralville, IA, USA). New England BioLabs restriction endonucleases and T4 ligase were used to carry out cloning. To perform all PCR reactions, GoTaq DNA polymerase (Promega, catalog# M3008) was used. To perform plasmid DNA isolation and the purification of gel fragments and PCR products, Qiagen products (QIAGEN, Chatsworth, CA, USA) were used.

### 2.2. Fish Husbandry

In this study, all procedures and treatments used on fish were approved by the Institutional Animal Care and Use Committee (IACUC), the University of Florida. Clinically healthy channel catfish (*Ictalurus punctatus*) fingerlings (2.5 ± 0.5 g) were purchased from Osage Catfisheries, MO, USA, and acclimated to laboratory conditions for two weeks. Fish were housed in 40-liter holding tanks (35 fish/tank) at a water temperature of 26 ± 2 °C. Instant sea salt and sodium bicarbonate were used to maintain a conductivity between 300 and 400 µS and pH between 7.0 and 7.4 of reverse osmosis (RO) water. Fish were fed with commercial pellets twice daily and kept on photoperiod at a 14 h of light:10 h of dark ratio. Fish were sedated with SYNCAINE^®^ (MS 222) (Nanaimo, BC, Canada) (40 mg/L) prior to being IC injected with either PBS or the vaccine.

### 2.3. Sequence Analysis

The unrooted phylogenetic tree of MurA was constructed via the “neighbor-joining” method using MEGA 6.0 software [22]. MurA protein sequences were retrieved from the publicly available NCBI GenBank database. *Salmonella enterica* (GenBank: EBI4772669.1), *Salmonella enterica* subsp. enterica serovar Typhimurium (EDL2719154.1), *Escherichia coli* (EFO4224361.1), *Shigella flexneri* (EFZ8852790.1), *Aeromonas hydrophila* (WP_025328540.1), *Aeromonas salmonicida* (HBL03892.1), *Edwardsiella hoshinae* (WP_024524569.1), *Edwardsiella tarda* (SPW31063.1), *Edwardsiella ictaluri* (WP_015870014.1), *Yersinia pestis* (PCN66959.1), *Vibrio anguillarum* (WP_026028554.1), *Vibrio cholerae* (WP_033932926.1), *Staphylococcus aureus* (NFZ33873.1) and *Bacillus cereus* (AUZ28172.1) were used. The Phyre2 web portal (http://www.sbg.bio.ic.ac.uk/phyre2/html/page.cgi?id=index) (accessed on 4 July 2023) was used to predict the three-dimensional (3D) structures of the *E. piscicida*, *E. ictaluri* and *S. typhimurium* MurA proteins.

### 2.4. Construction of E. piscicida Deletion–Insertion Mutation of *Δ*P_murA_

*E. piscicida* strain with the ΔP_murA180_::TT *araC* P_araBAD_ *murA* deletion–insertion mutation was constructed via PCR amplification of a 595-base pair DNA fragment upstream of the *murA* promoter in *E. piscicida* (J118) genomic DNA, using MurA1-SphI and MurA2-BglII primers (Table 2). The PCR-amplified fragment was inserted into pYA3700 [5], which was just upstream of the *araC* gene, in the SphI-BglII site. Primers pYA3700-FW and MurA2-BglII were used to verify correct plasmid isolates. A 499-base pair PCR fragment was amplified from J118 genomic DNA using primers MurA3-KpnI and MurA4-EcoRI. A modified Shine–Dalgarno (SD) sequence, known as “AGGAGG”, was tagged to the upstream primer MurA3-KpnI. The amplified fragment was inserted into the KpnI-EcoRI site of the above intermediate plasmid, and the resultant plasmid was confirmed via DNA sequencing. Using primers MurA1-SphI and MurA5-XmaI (Table 2), a 2441-base pair DNA fragment containing *araC* P_araBAD_ and *murA* 5′ and 3′ flanking regions was amplified from the latter intermediate plasmid and inserted into the SphI-XmaI site of the suicide vector pRE112 [20]. The recombinant plasmid was named pG8R8025 (Table 1) and confirmed via PCR amplification and restriction digestion with SphI-XmaI enzymes. The suicide plasmid pG8R8025 was conjugationally transferred from *Escherichia coli* χ7213 [18] to *E. piscicida* wild-type strains J118 to generate the ΔP_murA180_::TT *araC* P_araBAD_ *murA* mutant. Single-crossover strains were selected via Col and Cm-LB agar plates. The *sacB*-based sucrose counterselection was used to isolate mutants. The colonies derived from the sucrose plate were only screened for Cm^S^, Col^r^ and growth in presence of arabinose. Selected colonies were reconfirmed via PCR and sequencing. The *E. piscicida* strain containing ΔP_murA180_::TT *araC* P_araBAD_ *murA* mutation was named χ16016. 

### 2.5. Growth Curve Analysis

To obtain the growth curves of *E. piscicida* wild-type J118 and mutant ∆P_murA_ strains, overnight standing cultures of each strain were diluted 1:100 into pre-warmed broth and incubated at 30 °C while being shaken at 180 RPM. In order to compare the growth of the strains with and without arabinose, the broth used was either LB or LB plus arabinose (0.1% or 0.2%). Every 60 min, the OD_600_ values of the cultures were measured. The growth curves were calculated using the automated growth curve device Bioscreen C (Growth Curves USA, Piscataway, NJ, USA).

### 2.6. Attachment, Internalization and Intracellular Replication Assays

To identify the effect of ΔP_murA_ mutation on virulence, we compared the attachment, internalization and intracellular replication abilities of χ16016 to those of wild-type *E. piscicida* J118. 

Attachment, internalization and intracellular replication assays were performed as previously described [23,24]. Epithelioma papulosum cyprini (EPC) cells (ATCC, CRL-2872) were grown following the instructions given by ATCC. In brief, heat-inactivated fetal bovine serum (FBS) (Gibco BRL, Eggenstein, Germany) in eagle’s minimal essential medium (EMEM) (ATCC), 10% (*v*/*v*) was used to culture the cells. EPC cells were incubated at 30 °C supplied with 5% CO_2_. (A) Attachment assay: EPC cells were infected with J118 or χ16016 with or without arabinose at a multiplicity of infection (MOI) of 10. In order to reach an adequate infection level, the cells were incubated for 1 h. The monolayers were gently washed three times with cell culture medium to remove non-adherent *E. piscicida*. The cells were lysed with 1% Triton X-100 in PBS. The lysates were serially diluted in a 10-fold manner and plated on LB agar plates (with or without arabinose) to determine the adherence level. (B) Internalization assay: Internalized *E. piscicida* cells were counted using the gentamicin invasion assay. After infection, EPC cells were washed three times before incubation with 100 μg/mL of gentamicin for 1 h at 30 °C in a 5% CO_2_ incubator. After gentamicin treatment, cells were washed with PBS to remove the gentamicin and plated as described above. (C) Replication assay: After internalization, EPC cells were kept in growth media containing 5 μg/mL of gentamicin to eliminate extracellular *E. piscicida*. The intracellular bacterial population was assayed 2 and 4 h later. Cells were washed with PBS three times, and intracellular *E. piscicida* were calculated as described above.

### 2.7. Activation of TLRs and NLRs by E. piscicida *Δ*P_murA_ Strain 

We hypothesized that RAEVs with the regulated delayed lysis attribute could be used as an adjuvant to activate innate immunity by delivering highly conserved microbial structures, including lipopolysaccharide, flagella, CpG DNA, lipoprotein and peptidoglycan (PGN) fragments [diaminopimelic acid (DAP) and muramyl dipeptide (MDP)]. The detection of conserved microbial motifs relies on several classes of PRRs, including toll-like receptors (TLRs) and NOD-like receptors (NLRs) [25,26,27]. To examine the activation of TLRs and NLRs by χ16016, we utilized HEK-Blue cells expressing mouse TLR4, TLR5, TLR8, TLR9, NOD1 and NOD2 (all sourced from InvivoGen) with a NF_Κ_B-inducible secreted embryonic alkaline phosphatase (SEAP) reporter gene. We followed the protocol previously described by Swain et al., 2022 [9]. 

In brief, cells were maintained in Dulbecco’s modified eagle medium (DMEM) growth medium at 37 °C in 5% CO_2_. As recommended by the manufacturer, cells were cultured in the presence of selective antibiotics and passaged twice per week at 80–90% confluency. χ16016 and J118 cells were grown in LB broth with and without arabinose, respectively, to an OD_600_ of 0.8. HEK-Blue cells were subjected to infection with one of the two *E. piscicida* strains at an MOI of 1. Uninfected HEK-Blue cells were used as a control. SEAP activity was determined at 655 nm after 3, 6, 12 and 24 h of incubation. Triplicate wells were kept for each sample in order to yield significant results. TLR and NLR stimulation were expressed in terms of the SEAP activity level relative to that of control cells. 

### 2.8. Colonization and Lysis of χ16016 in Catfish Tissues

Catfish fingerlings were vaccinated with χ16016 via bath immersion for 2 h with 1 × 10^6^ CFU/mL. The colonization of χ16016 in kidneys and intestines at days 1, 2, 3 and 4 post-vaccination were evaluated as described previously [9]. During the lysis study, catfish fingerlings were IC injected with χ16016 or J118 at a dose of 1 × 10^3^ CFU/fish. Kidneys were collected from vaccinated fish every other day up to 16 days post-vaccination. Samples were collected from five fish in each time point, and the data consisted of a combination of three independent assays. The differences between two groups were analyzed via two-way ANOVA, where asterisks (*) indicated significant differences (** *p* < 0.01, **** *p* < 0.0001).

### 2.9. Determination of Lethal Dose 50 (LD_50_) χ16016 via Bath Immersion and IC Injection

To determine the LD_50_ of J118 and χ16016, catfish were IC injected with different CFU of bacteria (Table 3) in a 0.1-milliliter dose of BSG. In each treated group, there were 10 fish and two replicate tanks. Fish were observed twice daily for 21 days, and mortality was recorded. The LD_50_ values were calculated via the Reed and Muench method [28]. During the bath immersion, fish were immersed in tank water containing specific concentrations of bacteria ranging from 1 × 10^4^, 1 × 10^6^ to 1 × 10^8^ CFU ml^−1^, and there were 10 fish in each group (two replicate tanks). After 2 h, the fish were removed from the solution and placed into their original tanks. 

### 2.10. Evaluation of Anti-E. piscicida IgM in Catfish Serum and Skin Mucus via Enzyme-Linked Immunosorbent Assay (ELISA)

To assay antibodies in catfish serum and skin mucus, ELISA was performed using *E. piscicida* LPS following the protocol previously described [29]. In brief, *E. piscicida* LPS (100 ng/well) in coating buffer was used to coat the polystyrene 96-well flat-bottom microtiter plates (Dynatech Laboratories Inc., Chantilly, VA, USA). Wells were blocked by adding 300 μL of 5% bovine serum albumin (BSA). Diluted samples of catfish serum or 100 μL of mucus was added to wells in duplicate, while in blank control wells, 100 µL of sterile PBS was added. Moreover, 100 μL of diluted mouse anti-catfish IgM monoclonal antibody (Aquatic Diagnostics Ltd., Scotland, UK) was added to each well. Biotinylated goat anti-mouse IgG (Southern Biotechnology Associates, Birmingham, AL, USA) was diluted, and 100 μL of it was added to the wells. For the color development, *p*-nitrophenyl phosphate (PNPP, Thermo Fisher Scientific, Rockford, IL, USA) was added, and the OD was noted at 405 nm using an automated ELISA plate reader (model EL311SX; Biotek, Winooski, VT, USA).

### 2.11. Immunization and Challenge

Catfish fingerlings were divided into immunized (χ16016) and control (PBS) groups, and each group was split into triplicate tanks (n = 30 fish per tank). Fish were immunized using either PBS (control) or χ16016 (with dose 5 × 10^6^ CFU/mL) via bath immersion and marked as day 0. Different tissue samples i.e., gill, kidney, intestine, spleen and blood, were collected at 3, 5 and 7 days post-vaccination to measure the immune response via qRT-PCR. On day 14, fish were booster immunized using either PBS (control) or χ16016 with approximately same initial vaccination dose via bath immersion. On day 28, serum and skin mucus were collected (five fish from each group) to quantify the IgM via ELISA, and the fish were (both control and immunized) IC challenged with 3 × 10^5^ CFU of J118/fish. Fish were monitored for up to 21 days after challenge, and mortalities were recorded. Serum and skin mucus were collected (five fish from each group) on day 49 to quantify the IgM, and all of the surviving fish were humanely euthanized.

### 2.12. Isolation of RNA and Synthesis of First-Strand cDNA & Quantitative Real-Time PCR Analysis

The isolation of total RNA from gills, kidneys, intestines, spleens and blood was carried out using the TRIzol reagent (Invitrogen, Carlsbad, CA, USA) following the manufacturer’s protocol. The concentration and quality of isolated RNA samples were evaluated using a NanoDrop spectrophotometer. Total RNA was treated with 1 unit (U) of DNase I (Thermo Fisher Scientific) to eliminate genomic DNA contamination. First-strand cDNA synthesis was carried out using the oligodeoxythymine and random hexamer primer via the Thermo Scientific™ RevertAid™ Premium First Strand cDNA Synthesis Kit (Catalog # FERK1622). The cDNA synthesis was confirmed via the PCR amplification of the 18S rRNA gene. Quantitative real-time PCR (qRT-PCR) analyses of the target genes *il-8*, *il-1β*, *tnf-a*, *il-6*, *ifn-γ*, *cd4-1*, *cd4-2*, *cd8-α*, *cd8-β* and *mhc-ii*, as well as the reference gene 18S rRNA, were performed (Table 4) in Quantstudio 3 thermocycler (Applied Biosystems). The qRT-PCR reaction was performed in a 10-microliter reaction volume containing 1 µL of cDNA template, 0.25 µL of forward and reverse primers (2.5 mM each), 5 µL of 2 × PowerUp™ SYBR™ Green Master Mix (Thermofisher Catalog # A25742) and 3.5 µL of PCR-grade deionized water. The qRT-PCR was performed in triplicate wells under the following cycling conditions: pre-incubation at 95 °C for 10 min, followed by 45 cycles of denaturation at 94 °C for 10 s, annealing at 58 °C for 10 s and extension at 72 °C for 10 s, as well as a final step at 72 °C for 5 min. Negative control reactions were maintained without the template (cDNA). Melting curve analysis was carried out at the end to verify the integrity of the amplified products. The relative expression ratios were obtained by normalizing the expression of the target gene [mean crossing point (cp) deviation by that of a housekeeping gene-encoding 18S rRNA following the 2^−ΔΔCT^ method]. The results were expressed as the mean ± standard error (bars) of the three separate experiments. Differences between uninfected (control) and infected groups were analyzed via two-way ANOVA, where asterisks (*) indicated significant differences (* *p* < 0.05, ** *p* < 0.01, *** *p* < 0.001, **** *p* < 0.0001) with respect to the control group.

### 2.13. Statistical Analysis

Statistical analysis was performed via GraphPad Prism 6 (Graph Pad Software, Inc., San Diego, CA, USA). Survival data were analyzed using the log-rank (Mantel–Cox) test. Differences between the groups were analyzed via two-way ANOVA, where asterisks (*) indicated significant differences (* *p* < 0.05, ** *p* < 0.01, *** *p* < 0.001, **** *p* < 0.0001).

## 3. Results

### 3.1. Analysis of Sequences, Construction of Phylogenetic Tree and Generation of Homology 3D Structure of MurA

**Figure 1:**
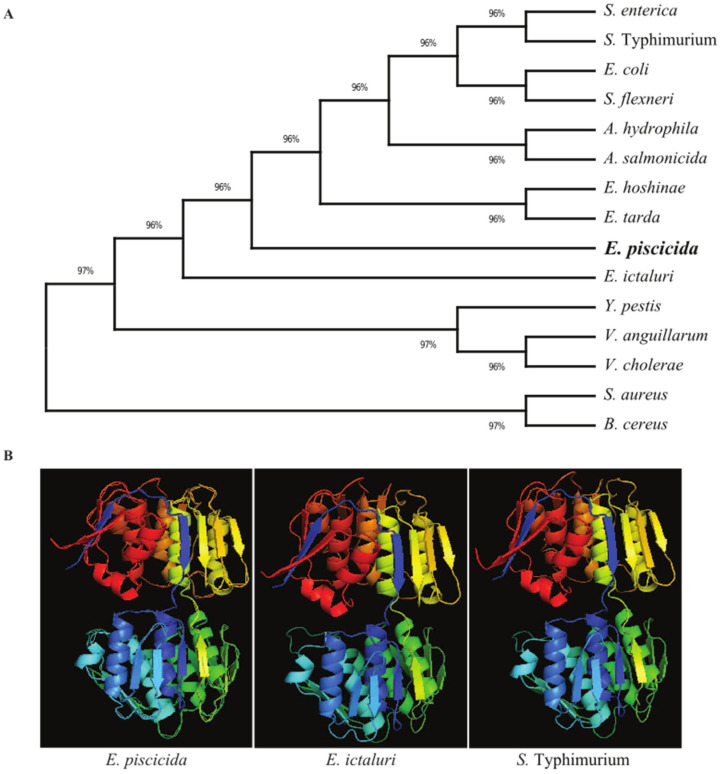
Evolutionary relationships and structural similarity between *E. piscicida* MurA and other bacteria. (**A**) Amino acid sequences of MurA of various bacteria were retrieved from the GenBank database, and the unrooted phylogenetic tree of MurA was inferred using the neighbor-joining method. The percentage of replicate trees in which the associated taxa clustered together in the bootstrap test (500 replicates) are shown next to the branches. Evolutionary analyses were conducted in MEGA X. (**B**) The 3D structure of the murA protein of *E. piscicida*, *E. ictaluri* and *S*. Typhimurium were predicted using the (PS) 2 Protein Structure Prediction Server Version 3.0 web portal.

The *E. piscicida* EIB202 (J118) *murA* open reading frame consists of 1257 base pairs (bp) that encode a putative 418-amino acid (aa) residue protein with an estimated molecular mass of 44.5 kilodaltons (kDa). In order to explore the evolutionary history of the bacterial MurA proteins, a phylogenetic tree was constructed using amino acid sequences (Figure 1A). The results revealed that the MurA proteins of *Edwardsiella* species share a high amino acid sequence identity, particularly those in *E. ictaluri* (98.80), *E. hoshinae* (97.37) and *E. tarda* (97.13). In the phylogenetic tree, the MurA proteins in *E. piscicida* and *E. ictaluri* form individual clusters. *E. hoshinae* and *E. tarda* MurA fall in the same cluster and are separated from other *Edwardsiella* species. *S. typhimurium* and *S. enterica* MurA proteins shared 87.88% sequence identity with the *E. piscicida* MurA protein and form a separate cluster to that of the *Edwardsiella* species. 

*E. piscicida*, *E. ictaluri* and *S. typhimurium* MurA 3D structures were generated using the Phyre2 web portal. Our results show that the MurA 3D structure is relatively conserved among these bacterial species. The MurA proteins in *E. piscicida*, *E. ictaluri* and *S. typhimurium* all have 14 α-helices and 21 β-sheets and, thus, share high sequence homology (Figure 1B).

### 3.2. Construction and Evaluation of E. piscicida murA Mutant

**Figure 2:**
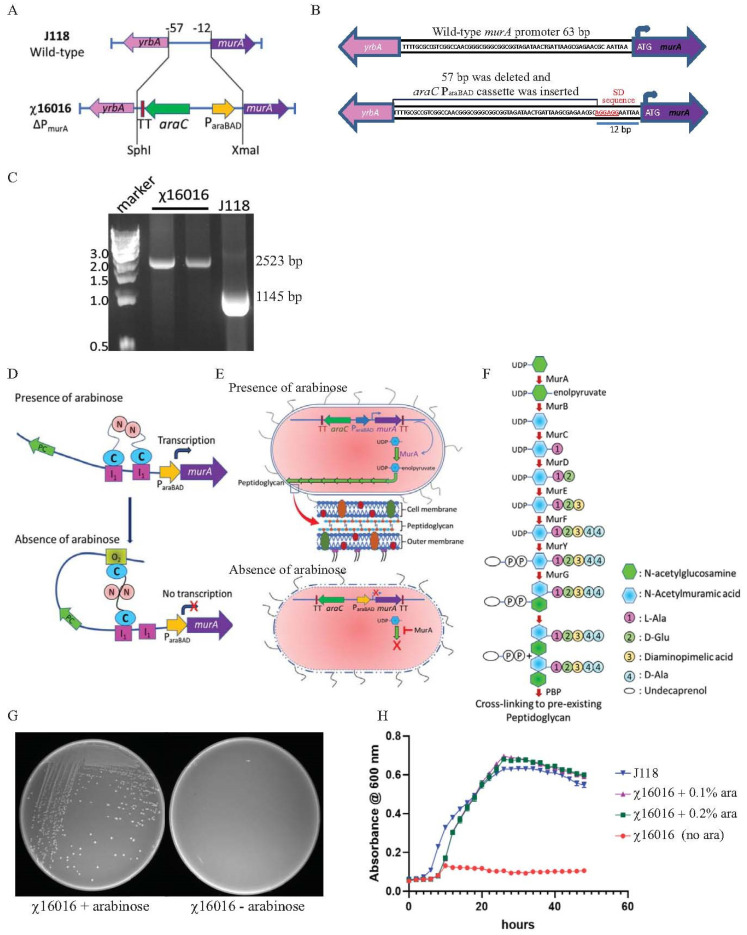
(**A**) Diagram of chromosomal deletioninsertion mutation of ΔPmurA: substitution of a tightly regulated araC ParaBAD cassette with the wild-type promoter of *E. piscicida* murA gene, resulting in arabinose-regulated murA expression. (**B**) Wild-type murA and ΔPmurA promoter region sequence: promoter of murA gene (57 bp) was deleted and replaced by araC ParaBAD cassette. Modified Shine–Dalgarno sequence is represented in red. (**C**) Genotype verification of ΔPmurA (χ16016). (**D**) Schematic illustration of arabinose regulation of chromosomal expression of murA gene. (**E**) Mechanisms of *E. piscicida* lysis systems: schematic representation of arabinose-dependent ΔPmurA (χ16016) cell wall synthesis. (**F**) Model of bacterial call wall peptidoglycan biosynthesis pathway; MurA catalyzes first step in peptidoglycan biosynthesis pathway. (**G**) Phenotypic characterization of ΔPmurA180::TT araC ParaBAD murA (χ16016) in presence or absence of arabinose in LB agar plates. (**H**) Growth curves for J118 and χ16016 in presence or absence of arabinose.

A conditional lethal *murA* mutant strain was constructed by replacing the chromosomal *murA* wild-type promoter of 57 bp (from −12 to −57 bp upstream from the start codon) with an *araC* P_araBAD_ cassette of 1335 bp. A modified Shine–Dalgarno (SD) sequence “AGGAGG” was introduced at −6 bp upstream from the *murA* start codon. The ΔP_murA180_::TT *araC* P_araBAD_ *murA* mutation was introduced into the wild-type *E. piscicida* strain J118, and the resultant strain was named χ16016. The chromosomal structure and sequence of the promoter region of the wild-type and χ16016 mutant strains are illustrated in Figure 2A,B. The ΔP_murA_ deletion–insertion mutant (χ16016) was confirmed via PCR using the primers MurA1-SphI and MurA5-XmaI. The replacement of the 57-base pair wild-type promoter by the *araC* P_araBAD_ promoter resulted in a DNA band that was larger than that of the wild-type sequence (Figure 2C).

### 3.3. Principle of Arabinose-Regulated MurA Synthesis 

In the presence of arabinose, the *AraC* protein changes conformation and forms a dimer that binds to I1 and I2 sites. This conformational change stimulates the transcription of *murA* (Figure 2D). In the absence of arabinose, the AraC protein dimer binds to the O_2_ and I1 regulatory regions on the *araC* P_araBAD_ promoter, generating a DNA loop that represses the transcription of the *araC* P_araBAD_ promoter and inhibits MurA protein synthesis (Figure 2D).

The bacterial peptidoglycan biosynthesis pathway is specified by a series of enzymes that have been widely studied [30]. MurA is involved in the first step of peptidoglycan synthesis within the bacterial cytoplasm, generating enolpyruvyl uridine diphosphate (UDP)-N-acetylglucosamine (GlcNAc) (Figure 1E,F). The deletion of *murA* is lethal since the product of the gene is a phosphorylated sugar that bacteria cannot take up from the environment. Therefore, replacing the chromosomal *murA* wild-type promoter with the *araC* P_araBAD_ activator promoter functions as a fitting conditional lethal mutation. The ΔP_murA180_::TT *araC* P_araBAD_ *murA* mutation was introduced into wild-type *E. piscicida* J118 to yield χ16016, and the phenotype of χ16016 was verified via growth in media with or without arabinose. In the absence of arabinose, the transcription of the *murA* gene was inhibited, and χ16016 was unable to grow. In the presence of arabinose, the growth was similar to that of J118 (wild type) (Figure 2G,H).

### 3.4. Attachment, Internalization and Persistence of E. piscicida Wild-Type and ΔP_murA_ (χ16016) Strains in Fish Cell Line

**Figure 3:**
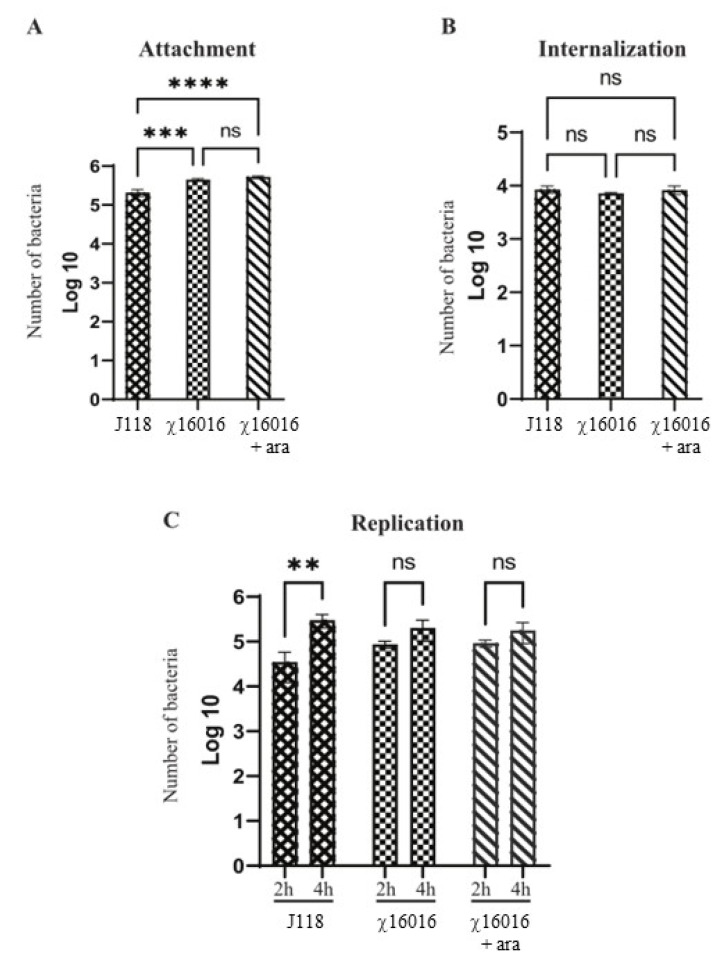
Abilities of *E. piscicida* wild-type and ΔP_murA_ (χ16016) strains to attach, internalize and replicate in fish cell lines. (**A**) Attachment assay: EPC cells were infected with J118 or χ16016 in presence or absence of arabinose at a multiplicity of infection (MOI) of 10 and incubated for 1 h to reach an adequate level of infection. Infected EPC cells were washed three times and lysed with 1% (*v*/*v*) Triton X-100 in PBS, and lysates were platted on LB agar plates with or without arabinose to assay the attached bacterial cells. (**B**) Internalization assay: After infection, the cells were incubated for another 1 h in cell culture medium containing gentamicin (100 mg/mL). Then, cells were lysed with 1% Triton X-100 in PBS, and lysates were plated on LB agar with or without arabinose plates to count internalized bacterial cells. (**C**) Replication assay: After gentamicin treatment, cells were incubated with growth media, and intracellular bacterial population was assayed two and four hours later. Cells were washed three times with PBS and lysed with 0.1 mL of 1% (*v*/*v*) Triton X-100 solution, and number of bacteria was assayed via the plating of lysates on LB agar with or without arabinose plate. The results are expressed as mean ± standard error (bars) based on three separate experiments. Differences between groups were analyzed via two-way ANOVA, where asterisks (*) indicated significant differences (** *p* < 0.01, *** *p* < 0.001, **** *p* < 0.0001, ns no significance).

The numbers of *E. piscicida* wild-type and mutant strain cells that attached, internalized and replicated in fish cell lines were assessed. Our results indicated that both wild-type and mutant strains proficiently attached to the EPC cells. However, the number of J118 that attached successfully was significantly lower than that of χ16016 with or without arabinose. There was no significant difference in the adherence ability between J118 and χ16016 with or without arabinose (Figure 3A). In the internalization study, there was no significant difference between J118 and χ16016 with or without arabinose (Figure 3B). There was no significant increase in the bacterial population between 2 and 4 h of incubation for χ16016 with or without arabinose, though there was a significant increase for J118 (Figure 3C). These results suggest that the MurA mutation somehow affects intracellular replication in EPC cells. 

### 3.5. Activation of NF-κB Pathway through TLRs and NLRs by E. piscicida Strains

**Figure 4:**
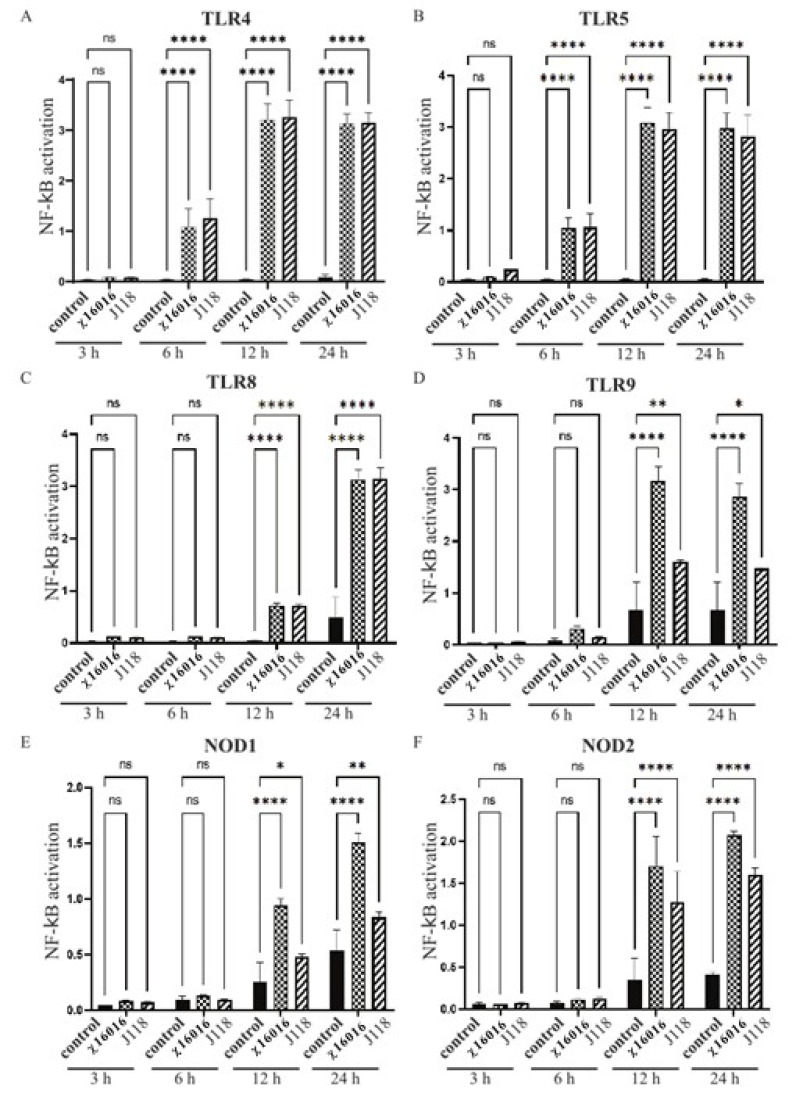
Activation of TLR4, TLR5, TLR8, TLR9, NOD1 and NOD2 by *E. piscicida* ΔP_murA_ (χ16016) strain. HEK-Blue^TM^ mTLR4 (**A**), mTLR5 (**B**), mTLR8 (**C**), mTLR9 (**D**), mNOD1 (**E**) and mNOD2 (**F**) were treated with χ16016 or wild-type strain J118 with an MOI of 1. After 3, 6, 12 and 24 h of incubation, secreted embryonic alkaline phosphatase (SEAP) activity was determined at 655 nm according to manufacturer’s recommendations (Invivogen). All samples were measured in triplicate. TLRs and NODs receptor stimulation was expressed relative to level of SEAP activity of untreated control cells. Differences between uninfected (control) and infected cell groups were analyzed via two-way ANOVA, where asterisks (*) indicate significant differences (* *p* < 0.05, ** *p* < 0.01, **** *p* < 0.0001 and ns no significance), with respect to the control cells.

The transcription factor NF-κB is involved in both innate and adaptive immune responses to various microbial infections, as well as the regulation of immune defense. TLR and NLR signaling leads to NF-κB activation and initiates inflammatory and antimicrobial responses to microbial infection. We analyzed the role that wild-type *E. piscicida* (J118) and ΔP_murA_ strains play in TLR and NLR signaling leading to NF-κB activation using HEK-Blue™-mTLR4, mTLR5, mTLR8, mTLR9, mNOD1 and mNOD2 cells. Our results indicated that the ΔP_murA_ mutant χ16016 was able to stimulate the TLR and NLR signaling pathways, thereby activating NF-κB at 6, 12 and 24 h post-infection. The levels of induction of TLR4-, TLR5- and TLR8-mediated NF-κB by χ16016 were similar to those of J118 (Figure 4A–C). The activation of TLR9-mediated NF-κB by χ16016 was significantly higher than that of J118 (Figure 4D). χ16016 also induced NOD1- and NOD2-mediated NF-κB to a higher degree than J118 (Figure 4E,F). 

### 3.6. Determination of Lethal Dose 50 (LD_50_) Dose of Wild-Type and Vaccine Candidate via IC Injection and Bath Immersion

Our results showed that the vaccine strain χ16016 injected via the IC route had a significantly higher LD_50_ (5 × 10^6^ CFU) than the wild-type strain (1.7 × 10^4^ CFU) in catfish (Table 3A). The LD_50_ of the wild-type strain J118 determined via bath immersion was 4.8 × 10^7^ CFU/mL. However, there were no mortalities in bath-immersed catfish at a high dose (4 × 10^8^ CFU/mL) of χ16016 (Table 3B).

### 3.7. Colonization and Lysis of χ16016 in Catfish Tissues

**Figure 5:**
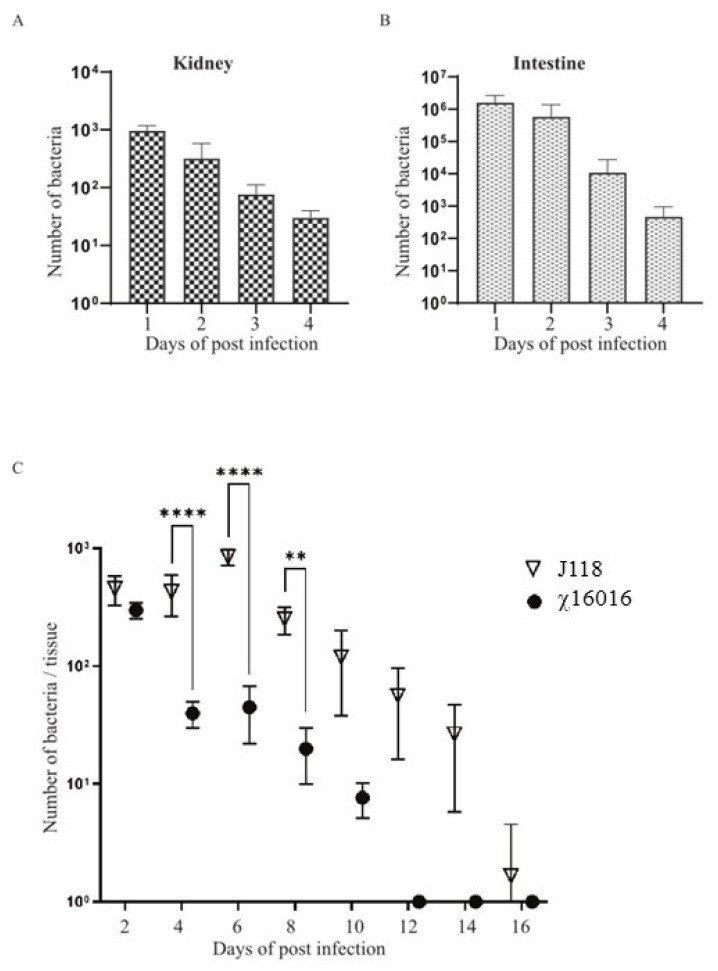
Colonization and lysis of χ16016 in catfish tissues. (**A**,**B**) Colonization of χ16016 in catfish kidneys and intestines at days 1, 2, 3 and 4 post-vaccination. (**C**) Lysis of vaccine strain χ16016 in catfish kidney. Differences between groups were analyzed via two-way ANOVA, where asterisks (*) indicate significant differences (** *p* < 0.01, **** *p* < 0.0001).

The ideal live attenuated bacterial vaccine should be sufficiently attenuated, delivered through a mucosal route and able to efficiently colonize fish tissues to elicit a potent immune response. To test the colonization efficiency of the ∆P_murA_ mutant delivered through bath immersion into the catfish tissues, kidneys and intestines were collected at days 1, 2, 3 and 4 from the catfish immunized with χ16016 via bath immersion. Significant numbers of bacteria colonized both the kidneys and intestines at 1, 2, 3 and 4 days post-vaccination (Figure 5A,B). This result indicates that the χ16016 successfully colonized and disseminated into different catfish tissues following bath immersion. To evaluate the cell lysis of χ16016 in fish tissue, catfish were IC injected with χ16016 or J118. Kidneys were collected from fish every other day up to 16 days post-vaccination. Both J118 and χ16016 significantly colonized catfish kidneys up to 2 days after vaccination. The number of χ16016 decreased with the progression of time and was completely lysed at day 12 in catfish tissues. The wild-type strain J118, however, persisted at day 16 (Figure 5C). This process ensures the complete safety of the vaccine and demonstrates the biocontainment property of the ∆P_murA_ strain. 

### 3.8. Expressional Modulation of il-8, il-1β, tnf-a, il-6 and ifn-γ Genes in χ16016-Vaccinated and Control Catfish

**Figure 6:**
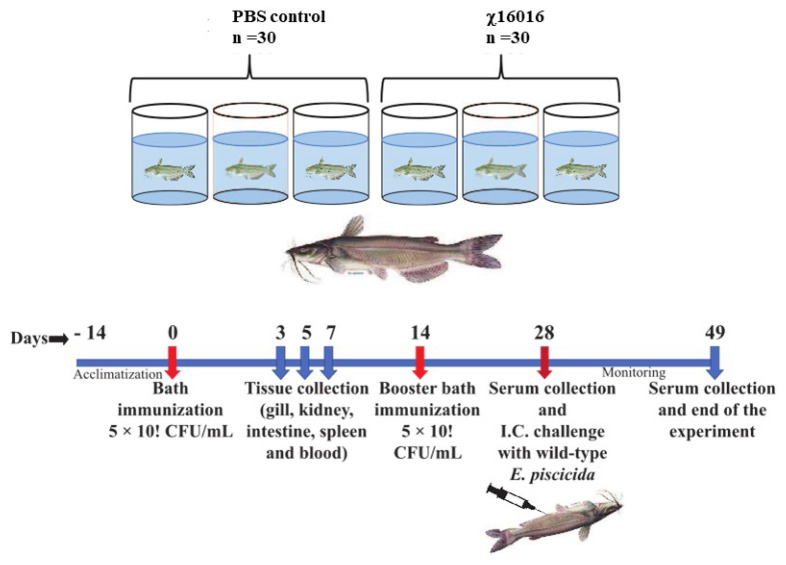
Summary diagram of the experimental setup used in the sampling of catfish.

**Figure 7:**
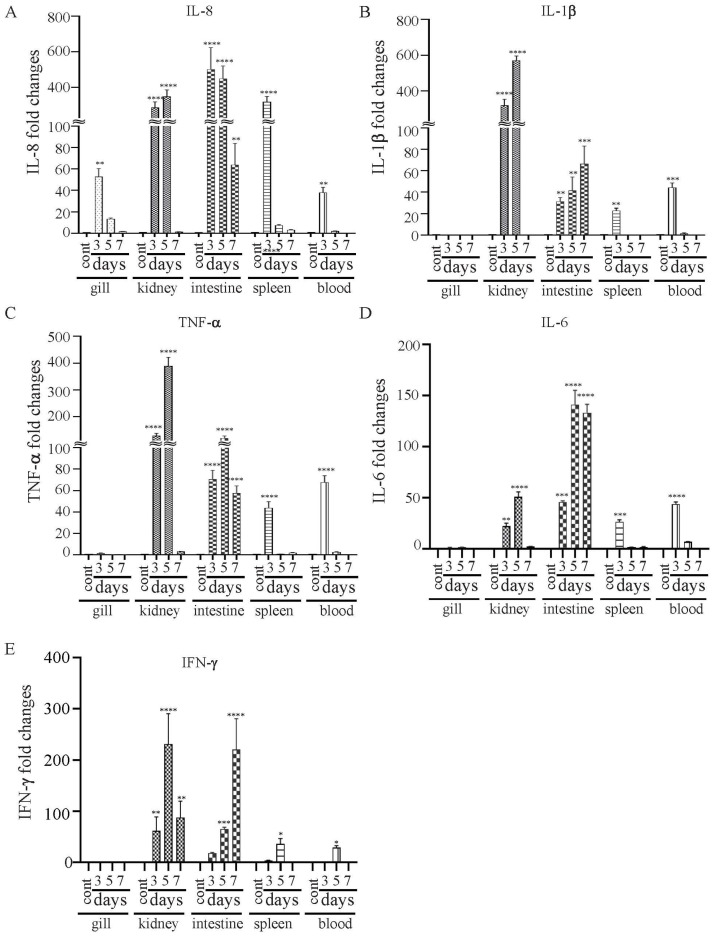
Changes in the expression patterns of *il-8* (**A**), *il-1β* (**B**), *tnf-a* (**C**), *il-6* (**D**) and *ifn-γ* (**E**) genes in χ16016-vaccinated and control catfish. Catfish were vaccinated with χ16016 via bath immersion. At 3, 5 and 7 days post-immunization, total RNA was extracted from the gills, kidneys, intestines, spleens and blood, and cDNA was prepared. The qRT-PCR assay was conducted to analyze the expression of *il-8*, *il-1β*, *tnf-a*, *il-6* and *ifn-γ* using 18S rRNA as an internal control. The results are expressed as mean ± standard error (bars) based on three separate experiments. Differences between uninfected (control) and infected groups were analyzed via two-way ANOVA, where asterisks (*) indicated significant differences (* *p* < 0.05, ** *p* < 0.01, *** *p* < 0.001, **** *p* < 0.0001) with respect to the control group.

The vaccine strain χ16016 has a biological containment system that is designed to cause programmed bacterial cell lysis upon the invasion of host tissues (in the absence of arabinose). We investigated its potential as a vaccine candidate to induce innate and adaptive immunity in catfish fingerlings. The summary diagram of the experimental setup and sampling outline are shown in Figure 6. The relative expression levels of cytokine genes *il-8*, *il-1β*, *tnf-a*, *il-6* and *ifn-γ* were assessed in the vaccinated and control catfish gills, kidneys, intestines, spleens and blood. The expression of *il-8* rapidly increased in all immunized fish tissues at days 3 and 5 compared to those of control fish tissues. The *il-8* gene was expressed to a higher degree in the intestine than in other tissues (Figure 7A). Significant induction of *il-1β* was noted in all of the tested tissues, except for the gills. The highest expression in the kidneys was detected at day 5 (~600-fold increase), the highest expression in the intestines was detected at day 7 (~70-fold increase), and the highest expression in the spleens and blood occurred on day 3 (~30- and 40-fold increase) (Figure 7B). *tnf-α* was expressed to a higher degree in the kidneys, intestines, spleens and blood of immunized fish than the control fish. At day 5, the highest expression was found in the kidneys (~400-fold increase) and intestines (~100-fold increase), whereas at day 3, elevated expression was noticed in the spleens (~50-fold) and blood (~70-fold) (Figure 7C). Increased expression of the *il-6* gene was detected in immunized fish intestines at days 3 (~50-fold increase), 5 (~160-fold increase) and 7 (~150-fold increase). Significant expression of *il-6* only occurred in the spleens (~25-fold) and blood (~50-fold) on day 3. In the kidneys, this expression was upregulated on both day 3 (~40-fold increase) and day 5 (~55-fold increase) (Figure 7C). *ifn-γ* showed very high expression levels in the intestines and kidneys. In the intestines, *ifn-γ* expression increased progressively on days 3 (~20-fold increase), 5 (~100-fold increase) and 7 (~300-fold increase). In the kidneys, *ifn-γ* expression was highest on day 5 (~300-fold), followed by day 7 (~120-fold increase) and day 3 (~100-fold increase). In the spleens and blood, *ifn-γ* expression only increased on day 5 (Figure 7D). 

### 3.9. Expressional Modulation of the T Cell-Related Genes in χ16016 Vaccinated and Control Catfish 

**Figure 8:**
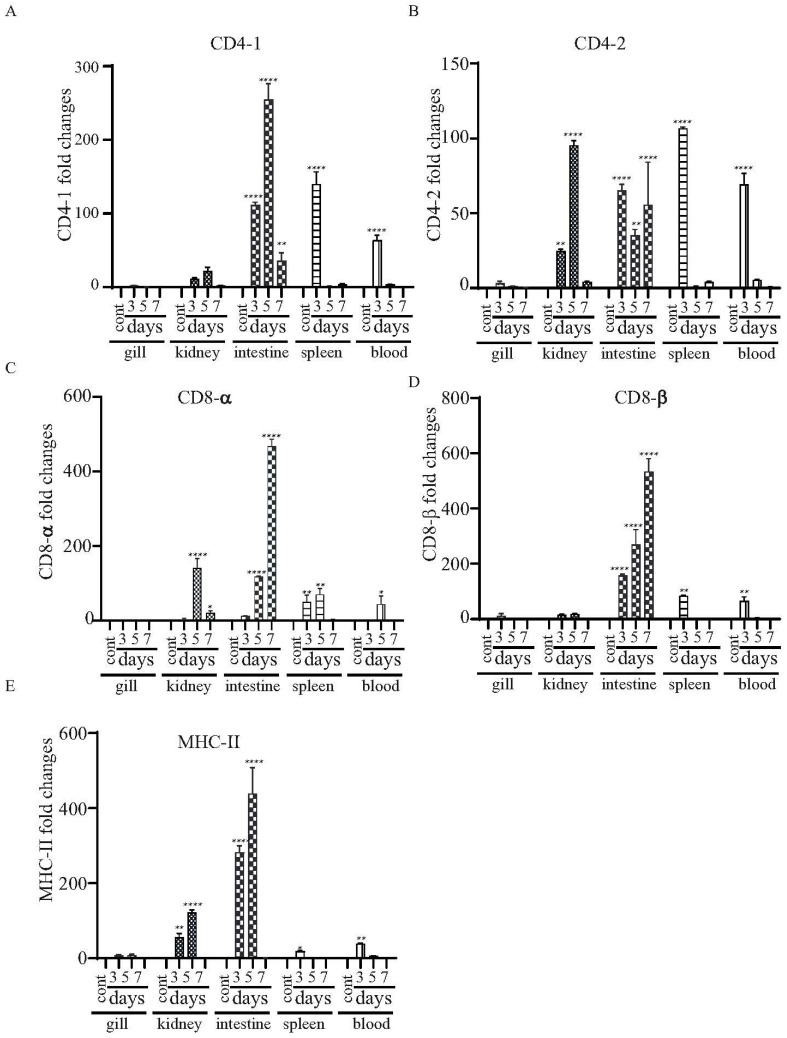
Changes in the expression patterns of the T cell- and B cell-related genes in χ16016-vaccinated and control catfish. Catfish were vaccinated with χ16016 via bath immersion. At 3, 5 and 7 days post-immunization, total RNA was extracted from the gills, kidneys, intestines, spleens and blood, and cDNA was prepared. The qRT-PCR assay was conducted to analyze the expression of *cd4-1* (**A**), *cd4-2* (**B**), *cd8-α* (**C**), *cd8-β* (**D**) and *mhc-II* (**E**) using 18S rRNA as an internal control. The results are expressed as mean ± standard error (bars) based on three separate experiments. Differences between uninfected (control) and infected groups were analyzed via two-way ANOVA, where asterisks (*) indicated significant differences (* *p* < 0.05, ** *p* < 0.01, **** *p* < 0.0001) with respect to the control group.

The major histocompatibility complex (MHC) molecules distinguish antigen-derived peptides from pathogens and display them on the cell surface to enable recognition by the relevant T cells in the acquired immune system. We evaluated the levels of mRNA expression in the *mhc-II* gene and T cell-specific genes (i.e., *cd4-1*, *cd4-2*, *cd8-α* and *cd8-β genes*), which are essential to driving adaptive immune responses upon vaccination. There was increased expression of both *cd4-1* and *cd4-2* in the kidneys and the intestines at all of the tested time points. However, enhanced expression in the spleens and blood was only detected at day 3 (Figure 8A,B). Both *cd8-α* and *cd8-β* were highly expressed in the intestines at days 3, 5 and 7, and expression increased continuously from day 3 to day 7 (Figure 8C,D). There was increased expression of *mhc-II* in the intestines and kidneys at days 3 and 5 (Figure 8E).

The function of MHC molecules is to bind peptide fragments derived from pathogens and display them on the cell surface for recognition by the appropriate T cells.

### 3.10. Analysis of E. piscicida Anti-LPS Antibody Responses in Control and Immunized Catfish

**Figure 9:**
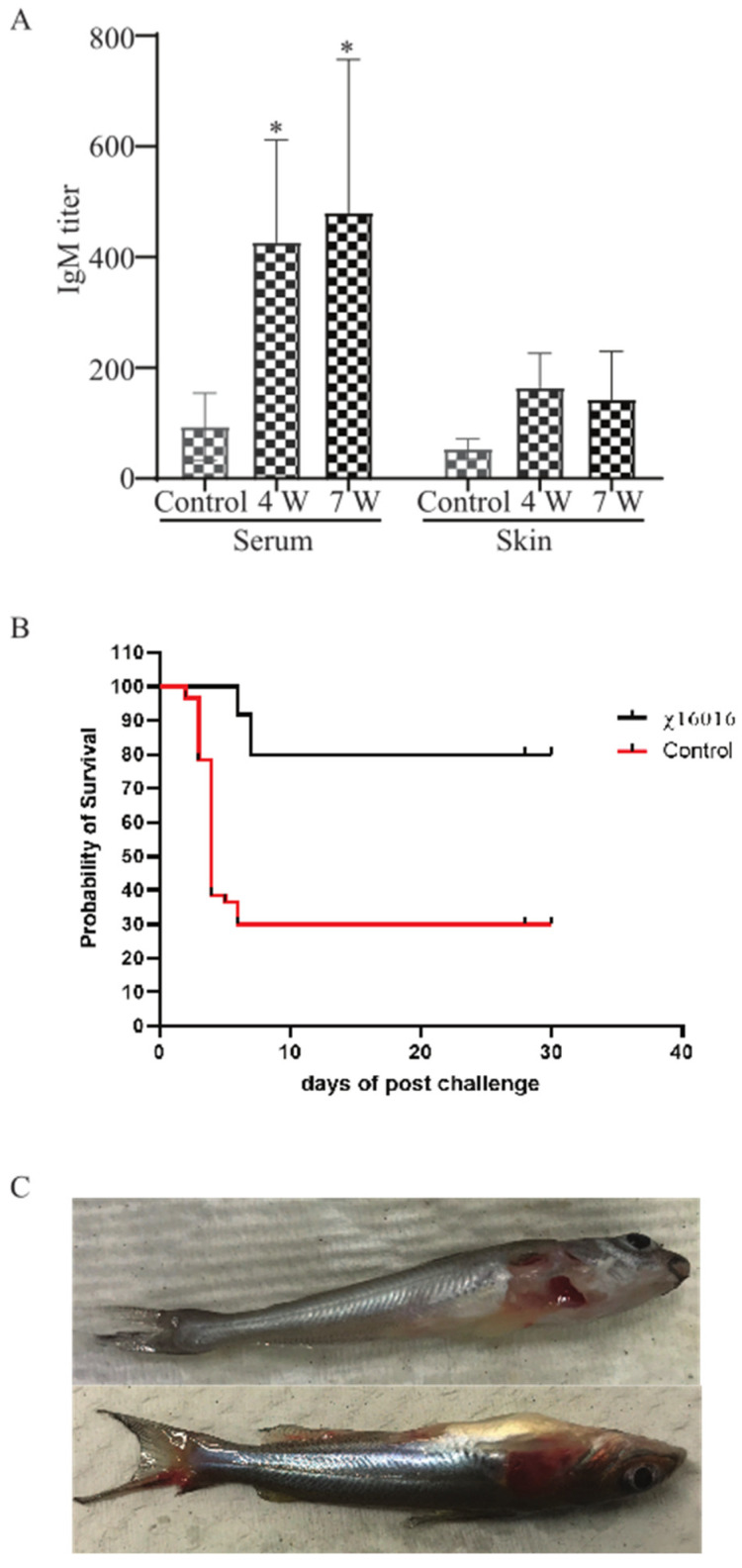
(**A**) Analysis of anti-LPS antibody responses in control and immunized catfish: Serum and mucosal immunoglobulin M (IgM) responses to *E. piscicida* lipopolysaccharide (LPS) were measured via ELISA at 4 weeks (4 w) post-vaccination with χ16016 and 3 weeks after J118 challenge (7 w) of immunized fish. Results are expressed as mean ± standard error (bars) based on three separate experiments. Differences between treated and control groups were analyzed via two-way ANOVA, where asterisks (*) indicated significant differences (* *p* < 0.05). (**B**) Survival rate of vaccinated fish after wild-type *E. piscicida* challenge: Control and 4 weeks post-vaccinated catfish were IC challenged with 3 × 105 CFU/dose of wild-type *E. piscicida* (J118). Mortality was recorded daily and represented as percentage rate of survival. (**C**) Clinical observation of catfish after challenge with *E. piscicida*.

*E. piscicida* lipopolysaccharide (LPS)-induced immunoglobulin M (IgM) in serum and mucus were measured via ELISA at 4 weeks (4 w) after vaccination with χ16016 and 3 weeks after the J118 challenge (7 w) of immunized fish. A significant increase in the IgM titer was detected in the serum of bath-immunized fish at weeks 4 and 7 compared to unimmunized control fish. The mucosal (skin) IgM titer steadily increased at weeks 4 and 7 in the immunized group (Figure 9A). 

### 3.11. Survival Rate of Vaccinated Fish after Wild-Type E. piscicida Challenge

To study the protective immunity induced by the vaccine strain χ16016, vaccinated catfish were challenged with the wild-type virulent *E. piscicida* strain J118. Our results indicated that χ16016 confers a greater rate of survival (80%) following a lethal challenge of wild-type *E. piscicida* than that of unvaccinated fish (30%) over a period of 4 weeks post-challenge (Figure 9B). 

### 3.12. The Clinical Observation of Catfish after Challenge with E. piscicida 

The majority of the mortalities occurred within 8 days post-challenge. Dead fish showed typical symptoms of edwardsiellosis, including hemorrhagic ulcers being present on the body’s surface and along the fins (Figure 9C). 

## 4. Discussion

The channel catfish (*Ictalurus punctatus*) contributes about 75% of the finfish aquaculture volume produced in the US and has a 35% value share, making it the main species produced in this industry. Edwardsiella piscicida, which is a Gram-negative intracellular pathogen, is responsible for a significant portion of losses in catfish aquaculture. Currently, vaccination is the most effective method of preventing and controlling infectious diseases in this industry [31,32]. A safe and highly efficacious vaccine is of utmost importance in terms of avoiding economic losses and improving food safety. An ideal vaccine for global fish aquaculture should be antibiotic-sensitive, highly immunogenic, environmentally safe, low cost and needle-free, as well as cause minimal stress during application [33,34]. Live vaccines possess the potent adjuvant properties required to elicit a high degree of immunogenicity, given that they are able to mimic natural infection and can be delivered via bath immersion or orally [10,35]. Live attenuated vaccines containing a defined deletion mutation can persist in the vaccinated animal and have the potential to be released into the environment. This process may lead to unintentional immunization of other species and may be problematic if these genetically modified organisms are able to persist in the environment [36,37]. To address this potential risk, the employment of biological containment systems is required. Our laboratory implemented a novel strategy to construct a biological containment system to prevent the spread of the vaccine strain into the environment by causing programmed bacterial cell lysis with no potential for survival [14,38,39]. Our recombinant attenuated *Edwardsiella* vaccine (RAEV) is the first live attenuated vaccine designed for use in the aquaculture industry that displays this biological containment property. The *murA* gene encodes enzymes involved in the first steps of peptidoglycan biosynthesis within the bacterial cytoplasm [40]. Since the *murA* gene produces a phosphorylated sugar that cannot be taken up by bacteria, *murA* deletion is lethal [41,42]. The murA protein in the fish pathogen *E. piscicida* is highly conserved and has a 3D structure similar to that of the well-studied human intracellular bacterial pathogen *Salmonella* sp. (Figure 1). In this study, a conditional lethal *murA* mutation was created by replacing the wild type *murA* promoter in *E. piscicida* with a tightly regulated *araC* P_araBAD_ promoter. This approach ensures that the expression of the *murA* gene is dependent on arabinose, which is provided during growth in vitro (Figure 2G,H). In the absence of arabinose, such as in fish tissues, muramic acid is not synthesized, peptidoglycan is not produced and lysis of the bacterium occurs [9]. Our lysis strain χ16016 has the ability to colonize fish tissues (Figure 5A,B), which is a requirement for the induction of a strong immune response [38], but it eventually dies due to lysis, demonstrating the biological containment feature (Figure 5C). The lysis strain has a similar capacity to attach and internalize into the fish cell line as the parental strain. However, its intracellular replication rate was slower than that of the wild-type, possibly due to the dilution of the pre-formed MurA enzyme with each round of cell division and the absence of arabinose inside of the cell (Figure 3). Vaccines with regulated lysis are excellent adjuvants; they efficiently stimulate innate immunity as the vaccine follows the natural infection process and, eventually, lyses to deliver ligands and stimulate pattern recognition receptors (PRRs). The lysis strain stimulates cell surface-expressed TLRs, such as TLR4 and TLR5, which are involved in the primary encounter between the pathogen and the host [43]. RAEVs deliver peptidoglycan subunits and nucleic acids upon lysis to activate intracellular PRRs NOD1, NOD2, TLR8 and TLR9. Our results demonstrate that lysis strain χ16016 stimulates TLRs and NLRs, leading to the activation of transcription factor NF-κB (Figure 4), which is important for the innate response and the establishment of the adaptive immune response [44,45]. Th1-type cytokines IL-1β, IFN-γ and TNF-α are involved in the management of intracellular infections [46,47]. IL-6 is involved in the maturation of B cells and the differentiation of macrophages. It is produced by antigen-presenting cells (APC), which include macrophages, dendritic cells and B cells [48]. Recombinant IL-6 has been used as an adjuvant in fish to induce cellular and humoral immunity, as well as to protect against *E. tarda* infection [49]. IL-8 plays an important role in the recruitment of T cells and non-specific inflammatory cells to the site of infection by activating neutrophils [50,51]. We observed the induced expression of *il-8*, *il-1β*, *tnf-α*, *il-6* and *ifn-γ* in the gills, kidneys, intestines, spleens and blood of χ16016 immunized catfish. In our previous study, we have shown that RAEVs are able to efficiently stimulate these cytokines [9,24,29]. These cytokine inductions may lead to the establishment of humoral immunity. In the adaptive immune response, major histocompatibility complex class II (MHC-II) molecules play critical roles in antigen presentation [52]. We observed significantly increased expression of *cd4*, *cd8* and *mhc-II* in the different organs of vaccinated catfish. These findings indicate the initiation of an adaptive immune response to *E. piscicida*. In teleosts, IgM is a primary antibody in humoral immunity. Since catfish do not have an IgT antibody isotype, IgM serves a vital function in both mucosal and systemic immune responses [53,54]. In immunized fish, measuring the IgM titer in response to the vaccine strain is an important parameter of adaptive immunity [55,56]. We observed that the vaccine-specific IgM antibody levels were greater in both the serum and skin of vaccinated fish than in that of the control fish. We assessed the immune protection mediated by the RAEV lysis vaccine strain χ16016 against *E. piscicida* infection and found that the survival rate was greater (80%) in the immunized group than in the control group (30%).

In summary, we have designed and constructed a recombinant attenuated *Edwardsiella* vaccine (RAEV) with a regulated lysis phenotype. It makes use of a biological containment system that results in programmed bacterial cell lysis, which prevents the vaccine strain from spreading into the environment. This lysis strain possesses the potent adjuvant properties required to stimulate the immune system and confers significant protection to catfish against wild-type *E. piscicida* infection. In conclusion, the ΔP_muA_ deletion–insertion mutation can be successfully used as a means of developing safe and effective bath immersion live attenuated vaccines for use in the aquaculture industry. This technology can also be used to develop an antigen or DNA vaccine delivery vector for use in the aquaculture industry.

## Figures and Tables

**Table 1:** Bacterial strains and plasmids.

Strain or Plasmid	Genotype and Relevant Characteristics	Source or Reference(s)
**Strains**		
*Escherichia coli*		
χ7213	*thi-1 thr-1 leuB6 glnV44 fhuA21 lacY1 recA1* RP4-2-Tc::Mu *λpir ∆asdA4 ∆zhf-2*::Tn*10*	[18]
*E. piscicida*		
J118	Wild-type *E. piscicida* EIB202, highly virulent, fish isolated, Col^r^	[19]
χ16016	J118 derivative; ΔP_murA180_::TT *araC* P_araBAD_ *murA*	This study
**Plasmids**		
pRE112	Suicide vector; *sacB mob*RP4 R6K *ori*; Cm^r^	[20]
pYA3700	Cloning vector containing TT *araC* P_araBAD_ cassette	[21]
pG8R8025	ΔP_murA180_::TT *araC* P_araBAD_ *murA*, pRE112	This study

**Table 2:** Primers used to perform ΔP_murA_ deletion–insertion mutation.

Primer	Sequence (5′-3′)
MurA1-SphI	F-CATGCATGCCGGGCATCACGTGTGTGGATATC
MurA2-BglII	R-CATAGATCTTCACAGACCGCTCAGCTTGCG
MurA3-KpnI	F-CATGGTACC AGGAGGAATTAAATGGATAAATTTCGT
MurA4-EcoRI	R-CCGGAATTCCGCTGACCTTGTCCATCACGATA
MurA5-XmaI	R-CATCCCGGGCGCTGACCTTGTCCATCACGATA
pYA3700	F-ATAACCTTTCATTCCCAGCGG

**Table 3:** (**A**) LD_50_ study of *E. piscicida* wild-type J118 and vaccine strain χ16016 in catfish via IC injection. (**B**) LD_50_ study of *E. piscicida* wild-type J118 and vaccine strain χ16016 in catfish via bath immersion.

(**A**)					
**J118 (Wild-Type *E. piscicida*)**					
**Dose** **CFU/Fish**	**Mortality**	**Deaths**	**Survivals**	**Mortality %**	**LD_50_**
9 × 10^2^	1/10	1	9	5	1.7 × 10^4^
9 × 10^3^	4/10	4	6	38	
9 × 10^4^	8/10	8	2	87	
9 × 10^5^	10/10	10	0	100	
9 × 10^6^	10/10	10	0	100	
χ16016 (ΔP_murA180_::TT *araC* P_araBAD_ *murA*)					
4 × 10^4^	0/10	0	10	0	5 × 10^6^
4 × 10^5^	0/10	0	10	0	
4 × 10^6^	4/10	4	6	40	
4 × 10^7^	10/10	10	0	100	
4 × 10^8^	10/10	10	0	100	
(**B**)					
**J118 (Wild-Type *E. piscicida*)** **CFU/mL**					
9 × 10^5^	0/10	0	10	0	4.8 × 10^7^
9 × 10^6^	2/10	2	8	20	
9 × 10^7^	10/10	10	0	100	
χ16016 (ΔP_murA180_::TT *araC* P_araBAD_ *murA*)					
4 × 10^6^	0/10	0	10	0	-
4 × 10^7^	0/10	0	10	0	
4 × 10^8^	0/10	0	10	0	

**Table 4:** Primers used to perform qRT-PCR assay.

Gene	Sequence 5′-3′	PCR Product Size in bp	GenBank #
18S rRNA	F-GAGAAACGGCTACCACATCC	128	AF021880.1
	R-GATACGCTCATTCCGATTACAG		
CD4-1	F-GGGGATTTTTGCACAGTTCGG	243	DQ435305.1
	R-TCCACAAGGTGATGCTGTTCA		
CD4-2	F-TGAACATCGGTGTTGTTGGTC	238	DQ435304.1
	R-TCCTGTTCAGTGAAGGTGACTT		
CD8-α	F-TCGTACAGCAAGACTAAGAAATCA	198	HQ446239.1
	R-TTTGATTGTTGTTGGGACTGTCT		
CD8-β	F-CTTAAGAACAGAGACACACCGGA	299	HQ446240.1
	R-ACACGCTTTGGATTTTTCACTG		
IL-1β	F-TGCTGGAAAGGTACTTTGACAG	296	DQ157743.1
	R-GCACTTCAAATTGATCACCATGC		
IL-8	F-GCTGCCCTTGATCATCTTTGC	219	AY145142.1
	R-GGATCCAGGCAGACCTTCATTC		
IFN-γ	F-TACTGGACATGAGATTCACAACCT	257	NM_001200217.1
	R-CTTTCACACTTTCCTGGAGTTC		
MHC-II	F-CACTGAAGCACAGATCAAACACC	236	AF103002.2
	R-CTGTTCTGTACACTCCTAGGTTGG		
TNF-α	F-TCTTCAGGAGTTTGGAGAACCTG	255	AJ417565.2
	R-CAGTTTCAAGCCTGAAGAGAAGG		
IL-6	F-GAAACATCTGGAGTCGAGCTGC	214	XM_017455306.1
	R-GACATTTTTGCGGGCTGAAGTC		

## Data Availability

Not applicable.

## Abbreviations

RAEV, recombinant attenuated *Edwardsiella piscicida* vaccine; murA, UDP-N-acetylglucosamine enolpyruvyl transferase; Δ, deletion–insertion; P, promoter; TT, transcription terminator; TLR, toll-like receptor; NOD1, nucleotide-binding oligomerization domain 1; NOD2, nucleotide-binding oligomerization domain 2. IL, interleukin; TNF, tumor necrosis factor; Fur, ferric uptake regulator; Crp, cyclic AMP receptor protein; DAP, diaminopimelic acid; IACUC, Institutional Animal Care and Use Committee; DMEM, Dulbecco’s modified eagle medium; SEAP, secreted embryonic alkaline phosphatase reporter gene; LD50, lethal dose 50; CFU, colony-forming unit; ELISA, enzyme-linked immunosorbent assay; BSA; bovine serum albumin; OD, optical density; cDNA, complementary DNA; MHC, major histocompatibility complex; LPS, lipopolysaccharide; IgM, immunoglobulin M; PRRs, pattern recognition receptors.
